# Facile Synthesis of Needle-like Copper Sulfide Structures as an Effective Electrochemical Sensor Material for Neurotransmitter Detection in Biological Samples

**DOI:** 10.3390/s23218849

**Published:** 2023-10-31

**Authors:** Aravindan Santhan, Kuo-Yuan Hwa

**Affiliations:** Department of Molecular Science and Engineering, National Taipei University of Technology, Taipei 10608, Taiwan

**Keywords:** Cu_2_S, needle-like structure, electrochemical sensors, serotonin, biological samples

## Abstract

Electrochemical sensors, due to their excellent and unique features, are of high interest nowadays for the detection and monitoring of several biological compounds. In such a case, serotonin (SRN), an important neurotransmitter, was herein studied for its detection in biological fluids since its presence is more crucial to be monitored and detected in clinical and medical applications. Several study strategies have been used to determine the chemical and physical properties. The crystalline size of the constructed copper sulfide (Cu_2_S) material was measured to be 25.92 nm. The Cu_2_S was fabricated over the working surface and further analyzed for several sensor parameters to be optimized. The charge transfer resistance of the copper sulfide-modified glassy carbon electrode (Cu_2_S/GCE) was determined to be about 277.0 Ω. With the linear range from about 0.029 μM to 607.6 μM for SRN, the limit of detection (LOD) was calculated as 3.2 nM, with a good sensitivity of 13.23 μA μM^−1^ cm^2^. The sensor experienced excellent repeatability, reproducibility, and long-term stability. The fabricated electrode was selective with the presence of different interfering compounds. The real sample analysis, as determined with the regular addition method with human serum and urine samples, revealed a good recovery percentage. Thus, the employed fabricated electrode material will be highly effective in sensing other analytes of choice.

## 1. Introduction

The neurotransmitter serotonin (SRN), also called 5-hydroxytryptamine, is mainly involved in certain functions, such as regulating neural activities [1]. SRN plays a major role in the regulation of neurogenesis and neuron elimination. SRN additionally regulates physiological processes, such as state of mind, sleep, hunger, and sexuality. The lack of SRN leads to several problems associated with our daily life, such as depressive symptoms, headaches, sexual disorders, carcinoid syndrome, sudden infant death syndrome (SIDS), and Parkinson’s disease. However, the excess of SRN also results in SRN syndromes and other related diseases [2,3,4,5]. Therefore, it is more critical to evaluate the concentration of SRN to detect the illnesses associated with the drug (SRN) and also to find and better understand the significance of SRN in neuron-related problems [6,7,8,9]**.** Identifying such illnesses in biological fluids and obtaining test results for a variety of conditions will be acceptable, utilizing extremely stable, reliable, and quick methods of detection. For SRN identification, a wide range of techniques, such as mass spectrometry, fluorescence spectrometry, SERS detection, enzyme immunoassays, and radioimmunoassay, are employed. However, when compared with the above and other approaches utilized, the electrochemical approach seems to be of greater significance, as SRN is electroactive and the study does not produce environmental damage. Aside from that, the electrochemical approach is less costly, requires less time, and does not require the pretreatment of samples in greater ratios.

The selection of material for modifying the working electrode is essential in electrochemical analysis, as it significantly participates in the oxidation of SRN and improves the electrochemical activity. Transition metal chalcogenides (TMCs) are attracting a significant amount of interest in a variety of areas, including electro-catalysis, optoelectronics, sensors/bio-sensors, medical science, energy conversion, and supercapacitors applications [10,11]. Among the TMCs, the metal sulfides, like molybdenum sulfides, tungsten sulfides, nickel sulfides, cobalt sulfides, iron sulfides, and copper sulfides, have been focused on and studied due to their distinctive properties and possible application [12,13,14,15]. Particularly, copper sulfide acts as a significant p-type semiconductor and has outstanding optical, electrical, and additional physiochemical characteristics. It has been thoroughly investigated for several applications, including photocatalysis, solar cells, electrocatalysis, catalysis, Li ion batteries, and sensors. Copper sulfides, which have a significant theoretical capacity (560 mAh g^−1^) as well as excellent electrical conductivity (10^−3^ S cm^−1^), have been studied as electrode materials for long time in electrochemical sensor applications [16,17,18,19,20,21,22,23,24,25].

Several reports on CuS with different micro/nano-structured morphologies have been published with the combination of carbon-based composites or any other hybrid composites for the electrochemical detection of compounds. Likewise, Chenghu Yuan et al. [26] reported a flower-like copper sulfide with boron and nitrogen co-doped carbon for the detection of nitrobenzene with excellent results. Yongfeng Chen et al. [27] prepared CuS with carboxylated carbon nanotubes (COOH-MWCNTs) formed as spherical particles under sub-micron size for the electrochemical detection of vanillin and tartrazine in foods. The obtained results were successful with the real-time detection in vanilla ice cream, with consistent results compared with the national standard method. Phumlani Tetyana et al. [28] prepared two different average-sized glutathione-capped copper sulfide nanoparticles for the electrochemical sensing of glucose. The temperature-dependent synthesis of copper sulfide showed good potential in real sample analysis in blood samples for blood glucose level real analysis. Also, Juan Peng et al. [29] studied copper sulfide nanoparticles decorated with graphene for alkaline phosphatase electrochemical sensing, showing outstanding results. Even though CuS can be prepared as nanoparticles or several other morphological structures, the electrochemical detection of analytes with/without composites shows excellent results. But, the preparation of CuS will be more efficient, easier, and cost-effective when compared with the hybrid combinations. Thus, a one-pot preparation to create CuS needle-like structures was employed in the present study.

Our present study investigated the electrochemical sensing of SRN using Cu_2_S. Initially, Cu_2_S was evaluated in order to analyze its physicochemical properties as well as its morphology. The electrochemical study began with the analysis of electrochemical factors, with the Cu_2_S modified over the glassy carbon electrode (GCE). The behavior of the developed electrodes under a variety of conditions, like optimization of Cu_2_S quantity, electrolyte optimization, and different concentration analysis of SRN and real-time research, were all studied. The hybrid synthesis of metal chalcogenides, such as Cu_2_S, will be less expensive and provide a precise and selective detection of SRN under diverse tests. 

## 2. Materials, Instruments, and Preparation

### 2.1. Materials and Instruments

The chemicals for the synthesis of copper sulfide were copper sulfate, thiourea, oxalic acid, and potassium hydroxide. Meanwhile, the chemicals used for the electrochemical sensing procedure were serotonin (SRN), dopamine, melatonin, epinephrine, uric acid, ascorbic acid, 4-nitrophenol, acetaminophen, chlorine ions, sodium ions, glucose, and hydroquinone. The materials and products, bought from Sigma-Aldrich, Taiwan, were directly utilized in the experiments without additional purifying processes. In cleaning and performing other studies, ethanol and distilled water were utilized. 

Characterization techniques, such as XRD, FTIR, Raman, XPS, FESEM, and EDAX, were carried out to explore the properties of Cu_2_S. A powdered sample of Cu_2_S was directly taken in the case of XRD and RAMAN analysis, which were studied with an X-ray diffractometer (PANalytical XPert PRO) with Cu-Kα radiation (λ = 0.1541 nm) and Raman Dong Woo 500 I (Korea) over the holder provided along with the instruments. For FTIR analysis, Cu_2_S powder was taken along with potassium bromide (KBr), which was ground and made into a pellet before exposing it for analysis. The prepared pellet was taken in the holder with FTIR—Fourier-transform infrared spectroscopy (FT-IR; Perkin Elmer CHI1000C, 6600)—and the FTIR spectrum of Cu_2_S was obtained, as shown below in the FTIR graph in the Results and Discussion section. For XPS analysis, the Cu_2_S sample was made in a suspension with DI water, sonicated (15 min), drop coated over an ITO (indium tin oxide) plate, dried in a hot oven, and then analyzed with XPS-ESCALAB 250, THERMO SCIENTIFIC Ltd., The Netherlands. For morphology and elemental analyses of the sample, Cu_2_S was dispersed in ethanol and sonicated for 15 to 20 min to obtain a colloidal solution. The sample was modified over the ITO (indium tin oxide) plate and then dried at 50 °C in a hot air oven. The as-modified sample over the ITO plate was sputtered and then analyzed via field emission scanning electron microscopy (FESEM) with a ZEISS Sigma 300 SEM microscope (morphology analysis), which was coupled with energy-dispersive X-ray spectroscopy (EDX) for elemental ratio analysis. The electrochemical studies were performed with cyclic voltammetry (CV) and differential pulse voltammetry (DPV) techniques with CHI6111E. The electrochemical setup with the three-electrode system was utilized, constituting the working electrode as a glassy carbon electrode (surface area—0.071 cm^2^); the reference electrode employed was the Ag/AgCl electrode, and the counter electrode was a platinum wire. For the electrochemical investigations, the sample preparation was initiated by dispersing the required Cu_2_S powder in 1 mL of DI water and then sonicated for 20 min. The suspension was later drop coated over the GCE surface for SRN detection.

### 2.2. Preparation of Copper Sulfide

**Scheme 1:**
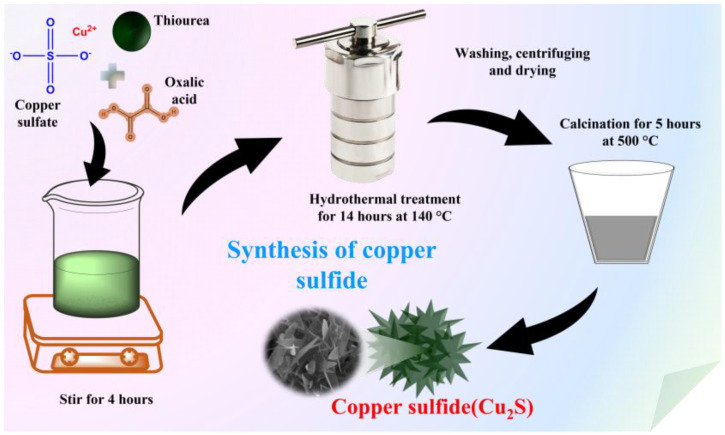
Synthesis scheme of Cu_2_S.

Copper sulfide was prepared by stirring about 0.2 M of copper sulfate and 0.2 M of thiourea in a beaker containing 75 mL of distilled water. Later, the aforementioned homogenous solution was mixed with 0.2 M of oxalic acid, followed with the addition of 0.2 M of potassium hydroxide. The mixture was mixed continuously at ambient temperature for 4 h after being stored at 140 °C for 16 h in a Teflon-coated autoclave [18]. The end product was centrifuged repeatedly using solvents like ethanol and distilled water at 9000 rpm for 20 min each. After centrifugation, the solid material was dried in a 50 °C hot air oven. After twelve hours of being dried, the final outcome was collected and calcined at 500 °C for 5 h in a muffle furnace [26,30]. The as-obtained powder of copper sulfide (Cu_2_S) was used for structural and morphological characterization and electrochemical applications for the detection of SRN. Scheme 1 gives a schematic overview of the synthesis procedure of Cu_2_S. 

### 2.3. Copper Sulfide Modified with a Glassy Carbon Electrode

The electrochemical monitoring of SRN was investigated using the GCE fabricated with Cu_2_S, so modifying the bare GCE should be done initially for SRN detection. The bare GCE was washed with ethanol and DW to remove the contaminants present over the working surface. Drop coating the washed GCE using the appropriate amount of Cu_2_S (3 mg—6 µL) and drying for 10 min at 50 °C was the next step. The same method was employed for all electrochemical variables’ analyses in sensing SRN.

### 2.4. Electrochemical Experimental Methods and Parameters

The electrochemical experimental methods involved cyclic voltammetry (CV) and differential pulse voltammetry (DPV). The electrochemical detection of SRN was studied with 0.01 M of SRN, with 0.1 M of phosphate buffer solution (pH–7) as the electrolyte. The scan rate was maintained constant at 0.05 V/s with the injection of 100 μM of SRN. The scan rate was varied in the case of the scan rate analysis, and SRN was varied during the concentration variation of SRN.

## 3. Results and Discussion

### 3.1. Structural Analysis

**Figure 1:**
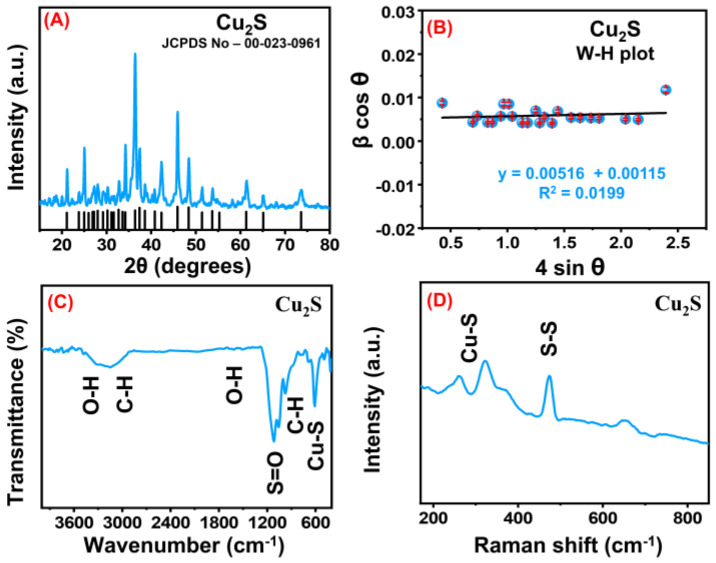
X–ray diffraction spectra, with stick patterns of Cu_2_S (**A**); W–H plot of Cu_2_S (**B**); FTIR spectra of Cu_2_S (**C**); and Raman spectra of Cu_2_S (**D**).

The XRD spectrum of copper sulfide (Cu_2_S) is shown in Figure 1A, exhibiting the diffraction peaks at 21.1°, 23.8°, 25.01°, 26°, 26.9°, 27.2°, 28.03°, 29.2°, 30.2°, 31.1°, 31.6°, 32.7°, 33.6°, 34.2°, 36.4°, 37.4°, 38.6°, 40.8°, 42.3°, 45.9°, 48.4°, 51.4°, 53.7°, 55.3°, 61.3°, 65.1°, and 73.6° correlating to the (222), (242), (302), (203), (180), (420), (262), (243), (091), (191), (134), (044), (224), (353), (433), (1 11 1), (174), (225), (444), (2 13 1), (2 14 0), (691), (406), (286), (486), (686), and (0 20 3) hkl planes, respectively, that can be attributed to Cu_2_S. Cu_2_S was identified via JCPDS card no. 00-023-0961, with orthorhombic crystal structures. A stick profile for Cu_2_S was clearly seen in the black line illustration, and the chemical composition confirmed that the sample has no contamination peaks apart from Cu_2_S. The average crystalline size of the as-prepared samples was calculated utilizing the as-determined formula as Scherer’s equation. The mean crystalline size (D), average dislocation density (δ), and average micro strain (ε) were identified from Equations (1)–(3) given below. Crystalline size:

(1) $$D=\frac{K\;\lambda }{\beta \;Cos\theta },$$

Micro strain:

(2) $$\varepsilon =\frac{\beta }{4\;tan\;\theta }$$

Dislocation density:

(3) $$\delta =\frac{1}{D^{2}}.$$

As a result, the outcomes acquired are displayed in Table 1. The crystalline size was determined via Scherer’s equations and the Williamson–Hall (W–H) graph, which indicated that the resultant grain size proved likewise comparable to that indicated in Table 1. The W–H plot, illustrated below, was additionally employed to determine the crystalline size for the developed materials. The resulting equation could be employed to identify the peak broadening and the strain-induced broadening:

**β_T_ = β_D_ + β_ε_** (4)

where β_T_ (peak broadening) = β_D (_broadening due to crystallite size) + β_ε_ (broadening due to strain).

From the Debye–Scherrer equation, we have:

(5) $$D=\frac{K\;\lambda }{\beta \;Cos\theta }\;(\mathrm{or})\;\beta_{D}=\frac{K\;\lambda }{D\;Cos\theta }$$

Similarly, the XRD peak broadening due to micro strain is given by:

(6) $$\beta_{\varepsilon }=4\;\varepsilon \;tan\;\theta$$

Equations (5) and (6) can be substituted in Equation (4) to provide:

(7) $$\beta_{T}=\frac{K\;\lambda }{D\;Cos\theta }+4\;\varepsilon \;tan\;\theta ,\;\beta_{T}\;Cos\theta =\varepsilon \;4sin\theta +\frac{K\;\lambda }{D}$$

Using Equation (7) as a straight line eq^n^, y = mx + c, we calculated the value of average crystallite size (given via y) and the lattice strain (given via the slope).

The W–H graph representing the compound is shown in Figure 1B as Cu_2_S. The above elaborated formulae were utilized in calculating the crystallographic size, employing the W–H diagram. It was noted that the mean crystalline size identified via the W–H approaches (26.87 nm) closely matching with the findings of Debye–Scherrer analysis (25.92 nm). Greater strain was considered to be the reason for the grain boundary action. From the aforementioned nanostructured materials’ category, the lesser crystalline size will lead to greater volumes of defects and vacancies. Considering that electrical conductivity might have an influence on electrochemical investigation, this method of analysis was proven to be more effective and appropriate. 

The as-prepared sample of Cu_2_S was further analyzed for the functional group analysis with FT-IR. Figure 1C displays the wavenumber spectrum between 400 cm^−1^ and 4000 cm^−1^. The bands that appeared at 3206 cm^−1^ and 3311 cm^−1^ were due to the C-H and O-H bond vibrations. The peak values around 610 cm^−1^, 880 cm^−1^, 1108 cm^−1^, 1341 cm^−1^, and 1640 cm^−1^ were attributed to the Cu-S, C-H, S=O, and O-H bond vibrations, respectively [31]. Thus, with the FTIR spectrum, the presence of Cu_2_S is evident. The Raman spectroscopy method was used to additionally investigate the Cu_2_S nanostructure materials, with a frequency range of 200 cm^−1^–800 cm^−1^, as illustrated in Figure 1D. In accordance with the literature review, 260 cm^−1^ and 474 cm^−1^ frequency ranges were identified as the S-S stretching vibrational mode of the A1g symmetric of S_2_ ions at the 4e sites, and this was evident due to the covellite phase of Cu-S nanostructures [32,33,34,35]. Therefore, the occurrence of Cu_2_S was also assured via the Raman spectra. 

**Figure 2:**
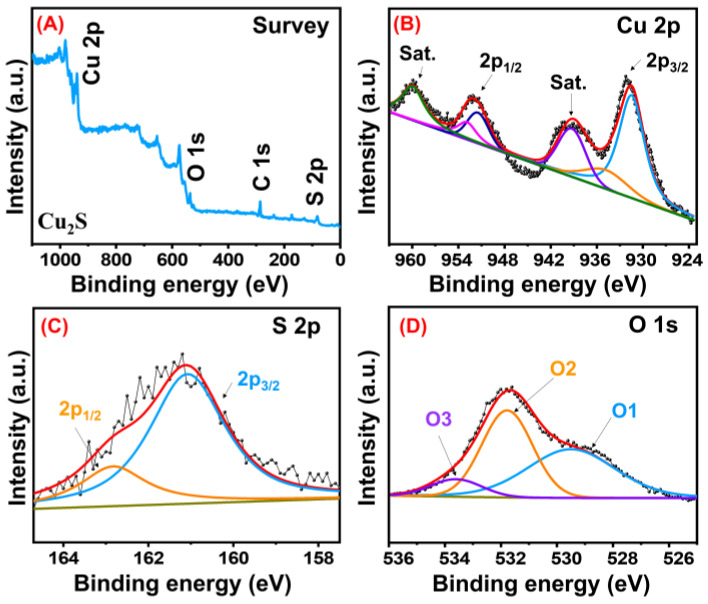
XPS overall survey (**A**); Cu 2p (**B**); S 2p (**C**); and O 1s (**D**).

Figure 2A–D shows the XPS analysis employed for determining the presence of Cu_2_S. Figure 2A depicts the entire XPS survey spectrum of Cu_2_S, which indicates its chemical presence as Cu 2p, S 2p, and O 1s. The presence of Cu 2p is shown in Figure 2B, which is deconvoluted at the binding energies (BEs) 932.5 eV and 952.2 eV. These binding energies were accredited to the two spin-orbital splitting events of Cu 2p_3/2_ and Cu 2p_1/2_, respectively. The remaining peaks were 939 eV and 959 eV, which represent the two satellite peaks [30,36,37,38]. The S 2p was fitted with two peaks at 161.1 eV and 163.0 eV, as shown in Figure 2C. The two peaks stated above were attributed to the two spin-orbital splitting events of S 2p_3/2_ and S 2p_1/2_, respectively, confirming the occurrence of S 2p [39,40,41]. The presence of oxygen, O 1s, was identified with the sample, which was deconvoluted at the BEs of 529.4 eV, 531.9 eV, and 533.4 eV, as shown in Figure 2D. These BEs were attributed to the lattice oxygen (M–O) at 529.4 eV, the hydroxide (M–OH) at 531.9 eV, and to the surface-adsorbed water molecules at 533.4 eV [30,41,42]. Therefore, the composition of the elements obtained from the XPS study shows the successful synthesis of Cu_2_S. 

### 3.2. Morphological Study

**Figure 3:**
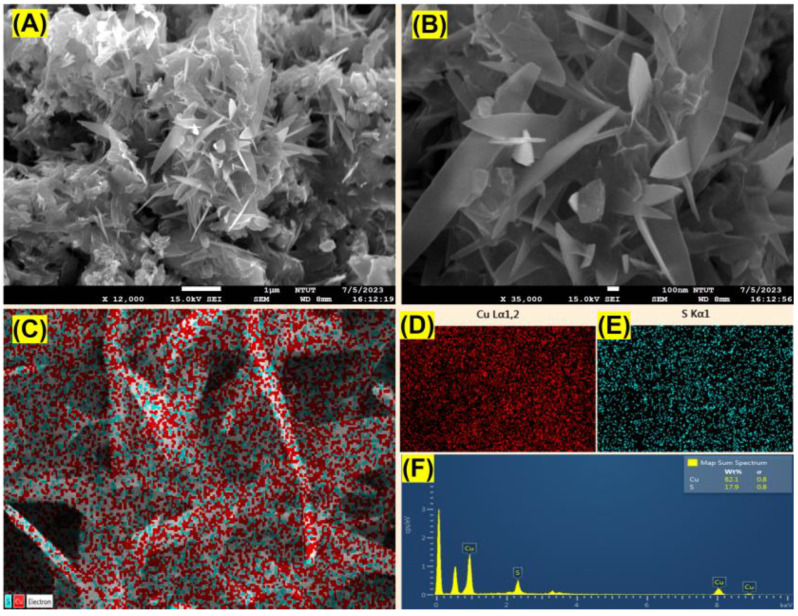
FESEM images with different magnifications of Cu_2_S (**A**,**B**), its respective elemental mapping (**C**–**E**), and EDAX spectrum of Cu_2_S (**F**).

The FESEM analysis of the Cu_2_S sample was analyzed, along with the EDAX and elemental mapping, to determine the morphology and the elemental presence in the sample. Figure 3A,B show the FESEM images of Cu_2_S under different magnifications as 1 µm and 100 nm. The Cu_2_S sample displays needle-like structured endings, as observed with the FESEM analysis for the Cu_2_S sample. It appears to be curved and bends at the center region, with the edges being thinner and smoother. Some non-uniform needles were also observed, which were attached, along with the uniform needle-like structures. The sharp edges initially resembled broader rods. Furthermore, the elemental mapping, as shown in Figure 3C–E, represents and confirms the elements as (C) a mix, (D) Cu, and (E) S. The EDAX spectrum of Cu_2_S is shown in Figure 3F. Thus, the presence of pure Cu_2_S with no other contaminants during sample preparation or during synthesis of the sample were identified, representing the purity of the prepared sample. 

## 4. Electrochemical Analysis

### 4.1. Electrochemical Analysis using Distinctly Modified Electrodes

**Figure 4:**
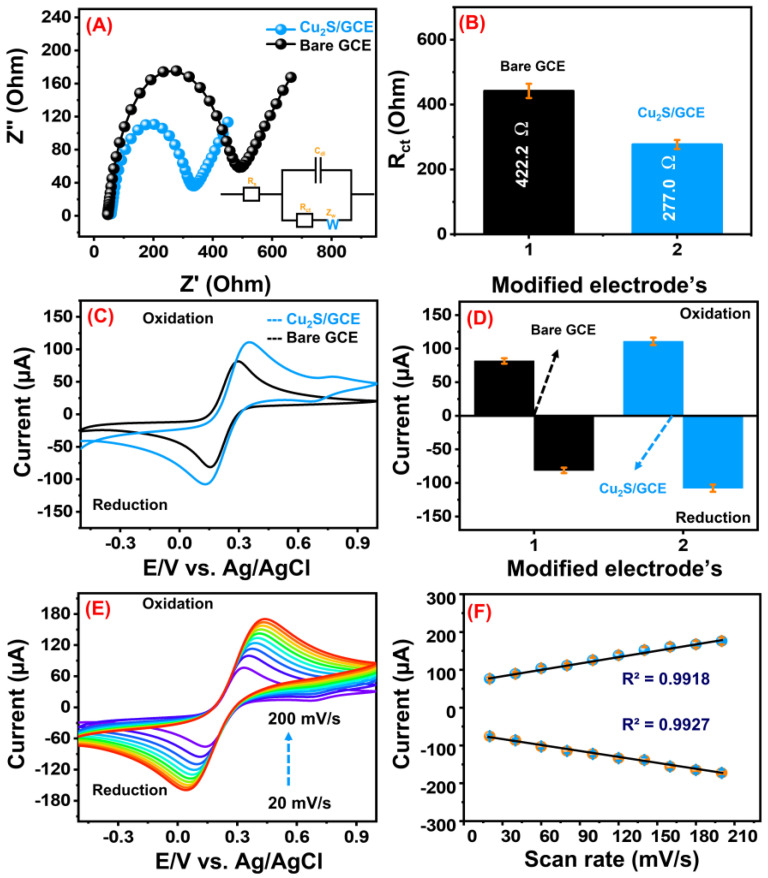
(**A**) shows the EIS profiles of the bare GCE and Cu_2_S/GCE, (**B**) histogram of EIS analysis, (**C**) cyclic voltammetry graphs of the bare GCE and Cu_2_S/GCE, with a scan rate of 50 mV/s, (**D**) its histogram plot, (**E**) CV profiles of scan rate analysis (from 20 mV/s to 200 mV/s) of the Cu_2_S/GCE, and its (**F**) corresponding linear plot of scan rate vs. current.

Electrochemical impedance spectroscopy (EIS) was employed to investigate electrodes’ impedance changes and to evaluate the electrical conductivity of the developed electrodes. The electron transfer resistance (Rct) values were mostly determined via the electrical and insulating characteristics of the electrolyte and electrode interface, and these can be calculated from the semi-circle diameters using Nyquist plots. Figure 4A shows the EIS profiles for the bare GCE, Cu_2_S/GCE obtained at 0.1 M KCl, and a mole ratio of 5 mM [Fe (CN)_6_]^3−/4−^, with the inset appearing as an equivalent circuit. The histogram of EIS analysis for different modified electrodes are shown in Figure 4B. According to the study outcomes, the bare GCE displayed a clearly stated semicircle owing to the higher frequency, with the obtained Rct values of 442.1 and 277.0 Ω for the bare GCE and Cu_2_S/GCE, respectively. The Cu_2_S/GCE had a significantly smaller Rct value of 277.0 Ω, which is drastically smaller than the bare GCE. This is because of the active interactions and distribution of Cu_2_S over the GCE’s surface representing an appropriate combination for evaluating the electrochemical performances. As a result of the EIS study, the Cu_2_S/GCE interface effectively interacted with the electrolyte employed, resulting in an excellent electron transfer rate attributable to the low Rct value. Followed with the EIS analysis, the surface area of the sample was studied with the cyclic voltammetry analysis (CV). This study was initiated in the presence of K_3_/K_4_ (5 mM [Fe (CN)6]^3−/4^) and 0.1 M of KCl under a 50 mV/s constant scan rate. The redox responses obtained for both the bare GCE and Cu_2_S/GCE are shown in Figure 4C, and the bar diagram for the CV analysis is shown in Figure 4D. The response for the bare GCE was Ipa = −81.6 μA, Ipc = −81.3 μA, Epa = 0.294 V, and Epc = 0.154 V. In contrast, the Cu_2_S/GCE experienced the following current responses: Ipa = 110.8 μA, Ipc = −107.9 μA, Epa = 0.350 V, and Epc = 0.129 V. The scan rate of the present study, which was kept constant, was varied from 20 mV/s to 200 mV/s, with the same electrolyte solution as K_3_/K_4_ and 0.1 M of KCl. The CV curves for the scan rate analysis is depicted in Figure 4E. The linear plot, as determined for the above study, is shown in Figure 4F, with the linear equations as Ipa = 564.29x + 68.253 and R^2^ = 0.9918, and Ipc = −536.88x − 67.665 and R^2^ =0.9927. The active surface area was analyzed with the Randles–Sevcik equation as follows:

I_p_ = (2.69 × 10^5^) n^3/2^AD^1/2^Cʋ^1/2^

where I_p_—the peak current, A—electroactive surface area, n—number of electrons transferred, D—diffusion coefficient, C—concentration of electrolyte solution, and ʋ—potential scan rate. The electroactive surface area was calculated to be 0.070 cm^2^ for the bare GCE and 0.098 cm^2^ for the Cu_2_S/GCE.

### 4.2. Electrochemical Analysis with the Presence of Serotonin (SRN)

**Figure 5:**
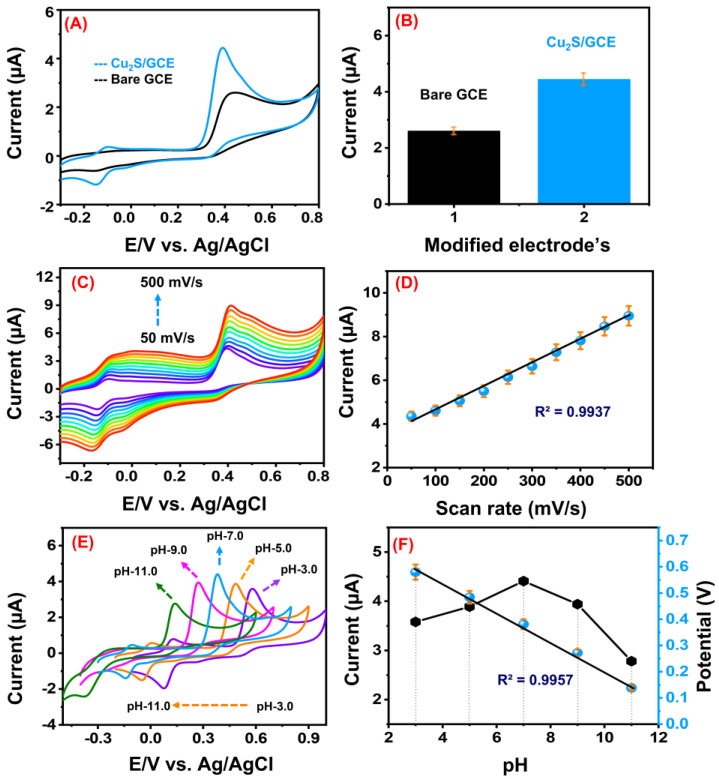
(**A**) CV study incorporating distinctly modified electrodes (bare GCE and Cu_2_S/GCE), with 100 μM of SRN in 0.1 M PB solution with a scan rate of 50 mV/s, (**B**) its histogram plot, (**C**) scan rate analysis studied at the Cu_2_S/GCE with 100 μM (SRN), (**D**) linear plot of various scan rates vs. currents (**E**) different pH levels studied with the same above conditions, and (**F**) linear plot of pH vs. current and potential.

**Scheme 2:**
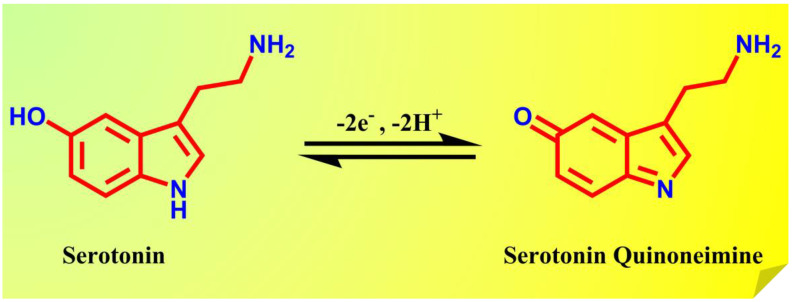
The serotonin electrochemical oxidation mechanism.

Initially, the different modified electrodes, namely the bare GEC and Cu_2_S/GCE, were studied for SRN detection in 0.1 PBS (pH 7.0) under a 50 mV/s scan rate and 100 μM of SRN, with the potential window ranging from −0.3 V to 0.8 V. The anodic peak current response for the bare GCE was 2.59 μA and 4.43 μA for the Cu_2_S/GCE, with the potential of 0.39 V. The observed changes are shown in Figure 5A, with the corresponding histogram shown in Figure 5B. The increased response of the Cu_2_S/GCE than the bare GCE was due to the higher conductivity of the material. The bare GCE was not sufficiently involved in the electrochemical oxidation of SRN when compared with the Cu_2_S/GCE. Also, the higher surface area of Cu_2_S was possibly coordinated with the response. Scheme 2 depicts the electrochemical oxidation of serotonin-to-serotonin quinone imine at the Cu_2_S-modified GCE. The oxidation process to serotonin quinone imine was clearly defined, with the presence of the oxidition response peak at 0.39 V. The Cu_2_S/GCE was analyzed under different optimization analyses, like the scan rate, pH optimization, and concentration of SRN, to better understand the Cu_2_S/GCE’s performance.

The scan rate of the study was varied from 50 mV/s to 500 mV/s at the Cu_2_S/GCE in 0.1 M PBS (pH 7.0), with the potential window from −0.3 V to 0.8 V, followed with the addition of 100 μM of SRN, as shown in Figure 5C. This study was conducted to know whether the scan rate has any influence over the study. The linear plot of different scan rates is shown in Figure 5D, with the linear equation determined to be Ipa = 10.668x + 3.5473 and R^2^ = 0.9937. The peak current response increases with the increase in the scan rate, as observed from the CV plot of the scan rate analysis. This increase in the peak current with respect to the scan rate was determined due to the participation of the reactants in the electrochemical reaction. At decreased scan rates, the reactants tend to diffuse at the electrode surface for a longer time. But, at higher scan rates, the rate of diffusion was lower, resulting in the low number of reactants to react with the electrode surface. These changes resulted with the changes in the peak currents as observed.

Next, the pH of the electrolyte (0.1 M PB solution) was optimized at the Cu_2_S/GCE, with the pH ranging from 3.0, 5.0, 7.0, 9.0, and 11.0, and performed under 50 mV/s scan rate with the injection of (100 μM) SRN. When studied at pH 3, the response was obtained at 3.58 µA, and the response was higher when the pH was changed to pH 5. The potential shift was also noticed, along with the higher peak current at 3.89 µA at 0.48 V. The pH was kept increasing to pH 7, whereas the peak response was even better than pH 5, as observed at 4.41 µA. However, the response declined when studied at pH 9 and pH 11, with the potential observed towards the more negative side. The response was higher at pH 7 rather than the other pH levels studied, which represents the optimum electrolyte condition for SRN to be studied. So, the optimized pH to be studied for SRN detection was pH 7 which was used for all other optimization analyses studied at the Cu_2_S/GCE. The CV curves of the pH optimization are shown in Figure 5E, with the linear plot with pH vs. potential and current shown in Figure 5F. The observed linear equation was y = −0.0548x + 0.7546 and R^2^ = 0.9957.

**Figure 6:**
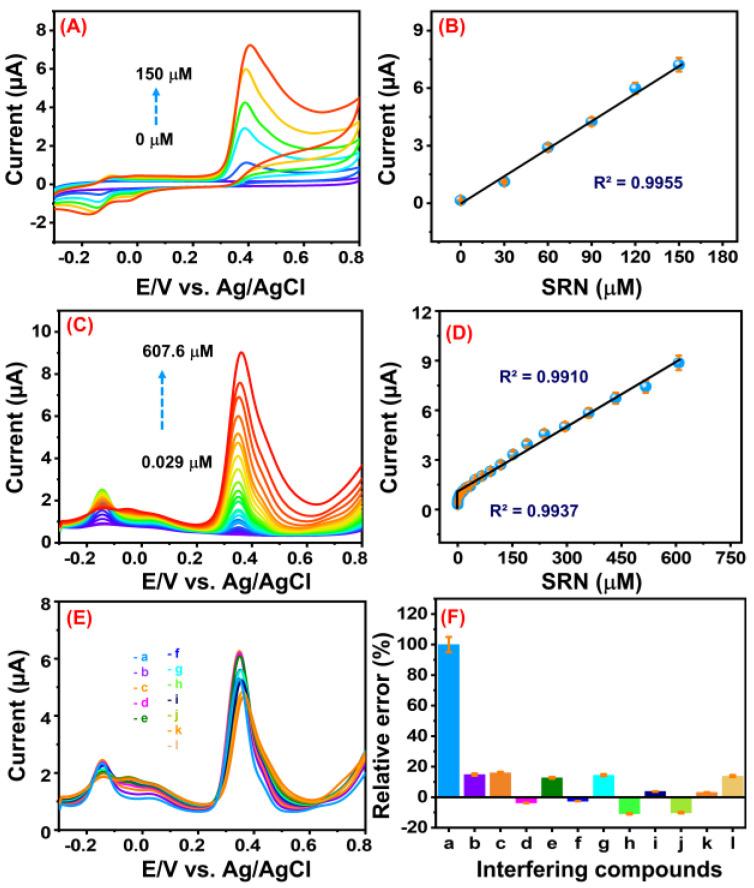
(**A**) Different concentrations (from 0 to 150 μM) of SRN under a scan rate of 50 mV/s studied with the Cu_2_S/GCE in the presence of a 0.1 M PB solution; (**B**) corresponding linear plot of different concentrations vs. current; (**C**) DPV plot of the Cu_2_S/GCE, with the addition of SRN (from 0.029 μM to 607.6 μM) in a 0.1 M PB solution; (**D**) calibration plot of SRN concentration vs. current; (**E**) selectivity analysis studied at the Cu_2_S/GCE, with 100 μM o SRN and with the presence of various interferents; and (**F**) relative error bars of the selectivity study.

The concentration of SRN varied from 0 μM to 150 μM at the Cu_2_S/GCE in 0.1 PBS pH 7.0, with a 50 mV/s scan rate at the potential window from −0.3 V to 0.8 V. The linear plot for the different concentrations of SRN is shown in Figure 6A. The CV curves show that the peak response increases along with the increase in the concentration of SRN. The Cu_2_S/GCE was stable at higher concentrations up to 150 µM, representing an excellent electrochemical performance of the modified electrode. The corresponding linear graph for the different concentration analyses is shown in Figure 6B. The linear equation was obtained as Ipa = 0.0489x − 0.0581 and R^2^ = 0.9955.

### 4.3. DPV Concentration Investigation

The DPV examinations were studied at the Cu_2_S/GCE interface for the most precise detection of SRN, and also to analyze and monitor the minor changes at different injections of SRN. The SRN concentration was successfully injected continuously, with a linear range from 0.029 μM to 607.6 μM in 0.1 M of PB solution with the same CV potential range. Figure 6C shows that the SRN injection has a constant rise in current responsiveness, with no variations in the potential. However, a high amount of SRN at the Cu_2_S/GCE interface caused minor changes in the DPV profiles. The linear equation employed from the continuous measurement arising with the SRN injection is represented, with two separate linear ranges representing the lower and higher concentrations of SRN. Figure 6D shows the calibration plot of SRN concentration vs. current. The linear equations and correlation coefficient for the lower and higher concentration injections of SRN are Ipa = 0.9267x + 0.2888 and R^2^ = 0.9937 at the lower concentration, and Ipa = 0.0132x + 1.0323 and R^2^ = 0.9910 at the higher concentration of SRN. The limit of detection (LOD) and sensitivity were calculated by evaluating the as-determined DPV graphs using the equation from the linear plot. The LOD is used in sensor technology to determine the injected concentration of analytes chosen. The formula was LOD = 3SD/σ; the standard deviation obtained for the performed analysis was denoted as SD, while the slope value of the calibration plot was denoted as σ, and both were employed to calculate the limit of detection of the study. The LOD was 3.2 nM for SRN sensing, with the sensitivity determined to be 13.23 μA μM^−1^ cm^2^. A table of comparison in Table 2 was illustrated to determine the performance of the constructed sensor over the previous SRN sensing reports. The outcomes obtained demonstrate that the Cu_2_S material boosted the electrocatalytic efficiency at the surface of the GCE for the efficient sensing of SRN.

### 4.4. Selectivity Analysis

When examined for various studies, the Cu_2_S/GCE confirmed all of the key characteristics and provided excellent outcomes for SRN detection. Nevertheless, the newly developed electrode’s selectivity is an essential sensing property to be studied. The selectivity study was conducted with the presence of different kinds of compounds categorized as interfering, anti-interfering, similar families, and closely related categories. With the DPV technique, all of the above-described compounds were examined using the same electrochemical setup, with the injection of SRN and other interfering compounds chosen. The DPV graphs acquired for the selectivity analysis are depicted in Figure 6E. Interfering compounds, such as dopamine (b), melatonin (c), epinephrine (d), uric acid (e), ascorbic acid (f), 4-nitrophenol (g), acetaminophen (h), chlorine ions (i), sodium ions (j), glucose (k), and hydroquinone (l), were regularly provided at a level that was 5-fold greater than SRN (a). The collected outcomes undoubtedly indicated that the Cu_2_S/GCE exhibits strong selective actions at lower levels than the other tested compounds. From the above examination, the major impact of potential is an essential factor, and the Cu_2_S/GCE is highly selective to SRN, even at 5-fold lower than the other interfering agents added. Figure 6F shows the relative error plot for the selectivity study analyzed. Thus, the Cu_2_S/GCE is superior, exhibiting the majority of the properties suitable for the electrochemical investigation of SRN with excellent results.

### 4.5. Electrochemical Sensor Parameter Analysis

**Figure 7:**
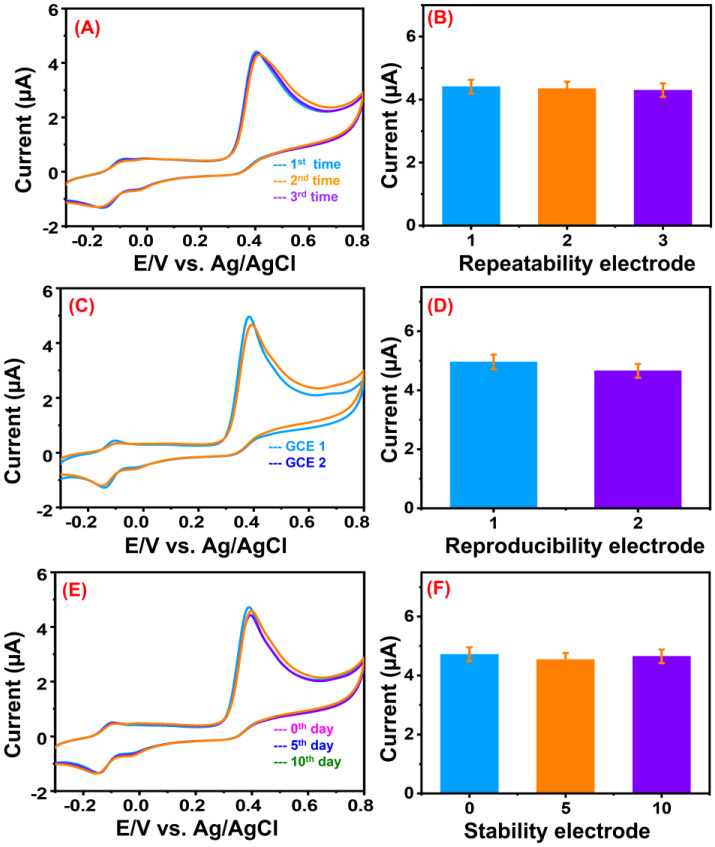
(**A**) Repeatability, (**C**) reproducibility, (**E**) stability, and its (**B**,**D**,**F**) corresponding histogram plots.

The other electrochemical investigations were carried out to demonstrate an electrode’s distinctive characteristics under various conditions, including the repeatability, reproducibility, and stability. All of these analyses were studied under similar conditions, involving the use of 0.1 M of PB solution (pH 7), SRN (100 µM), and a 50 mV/s scan rate. The repeatability experiment was evaluated for almost three consecutive studies over SRN detection at the Cu_2_S/GCE interface. Figure 7A displays the CV profiles recorded for the repeatability investigation following the injection of (100 μM) SRN. The Cu_2_S/GCE was repeatedly reflecting the constant oxidation response for SRN sensing. The CV analysis was conducted to determine the reproducibility of Cu_2_S, which was studied with two different electrodes. Figure 7C displays the aforesaid investigation, employing the same conditions used in the repeatability experiment with the change in the working electrode. Only tiny differences were observable, with the oxidation of SRN showing similar responses in each of the two independent GCEs. The stability study was demonstrated over ten consecutive days, having a 5-day break in between till the tenth day. The CV plot was obtained for the stability analysis with the Cu_2_S-modified GCE, as shown in Figure 7E. The decreased response was fairly constant throughout all ten days of measurements, despite only a tiny alteration in the anodic current response. This phenomenon was related to the surrounding circumstances during the experiment. Figure 7B,D,F illustrates the histogram analysis of repeatability, reproducibility, and stability.

### 4.6. Real Sample Analysis

**Figure 8:**
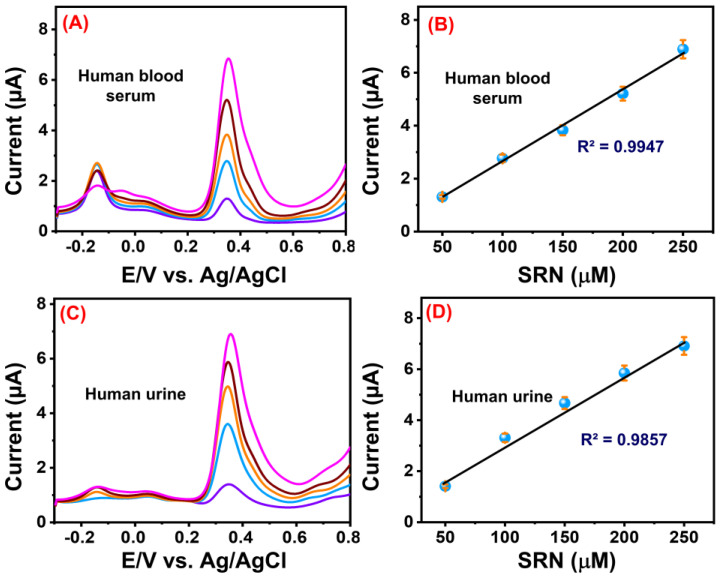
(**A**,**C**) DPV curves of real sample analysis performed at the Cu_2_S/GCE surface with (**A**,**C**) human blood serum and human urine samples, with their (**B**,**D**) corresponding linear plots.

The most significant outcomes for SRN monitoring were acceptable; therefore, the Cu_2_S/GCE was validated on real samples. SRN was found to be of greater significance following real samples of human blood serum and human urine. All of the collected real samples have been preconditioned before DPV testing. The investigation employed for real samples were performed following the proper regulations and laws, as observed in accordance with the guidelines of the institutional review board (IRB) IRB202101EM003 provided to Dr. Hwa. Each diluted sample was taken for DPV analysis conducted at the Cu_2_S/GCE, employing the regular addition procedure using SRN. The DPV curves for the analysis are shown in Figure 8A,C and the linear images are given in Figure 8B,D, with the appropriate correlation coefficients given as the inset. Moreover, the acquired data showed that SRN detection over the Cu_2_S/GCE surface was beneficial, with significant recovery rates.

## 5. Conclusions

The Cu_2_S combination was developed for studying the SRN sensor application, which showed a high level of responsiveness. The results of the structure and morphological investigations showed that the samples were more effectively present, with no other impurities. The various optimization analysis, like varied scan rate, pH, and concentration of the Cu_2_S and SRN, was studied and the optimum concentration was chosen showing higher electrochemical performances. The Cu_2_S/GCE surface for SRN monitoring exhibited improved responses, having two distinct linear ranges and a lower detection limit of about 3.2 nM, with good sensitivity. The repeatability analysis of the fabricated electrode showed a constant response for repeated analyses of SRN. The stability study performed for a period of ten days also had similar responses when compared with the initial day of analysis. The reproducibility analysis showed the fabricated electrodes’ reproducible behavior, even following the modification of independent electrodes. The sensor’s selectivity was highly selective, even with the presence of various interferent analytes used. The recovery outcomes for real samples studied in biological samples were excellent, having good recovery percentages. The synergistic reaction occurring on the Cu_2_S surface enhanced the oxidation by improving its current responses. Thus, Cu_2_S will be an efficient and effective electrocatalyst in detecting SRN, and other applications can also be studied, taking in account of Cu_2_S’s activity.

## Figures and Tables

**Table 1:** The mean crystalline size was obtained via the Scherrer equation and compared with the W–H approach, along with the average strain and average dislocation density.

Prepared Nanomaterial	The Mean Crystal Size D (nm): Scherrer Formula	The Mean Crystal Size D (nm): W–H Approaches	The Average Micro Strain (×10^−3^)	The Average Dislocation Density δ
Cu_2_S	25.92	26.87	4.98	4.56

**Table 2:** A comparison table representing the distinctly modified electrodes of previous reports for SRN detection.

Material	Method	Linear Range (µM)	Limit of Detection (µM)	Ref.
Pt/MWCNT/PPy/AuNPs	DPV	0.50–5.0	0.15	[6]
rGO-Co_3_O_4_	Amperometry (IT)	1–10	1.128	[43]
rGO-Ag_2_Se/GCE	DPV	0.1–15	0.029	[2]
rGO-AuNPs	SWV	0.4–10	0.04	[44]
ZIF-67/MWCNT	Amperometry (IT)	0.049–800	0.007	[5]
Poly (FSBF)MPGE	CV	10–50	1.7	[45]
Cu_2_S/GCE	DPV	0.029 to 607.6	0.0032	This work

Pt/MWCNT/PPy/AuNPs—MWCNTs polypyrrole and colloidal silver nanoparticles on the platinum electrode, rGO-Co_3_O_4_—cobalt oxide nanocubes with reduced graphene oxide, rGO-Ag_2_Se/GCE—silver selenide on reduced graphene oxide, rGO-AuNPs—reduced graphene oxide—gold nanoparticles, ZIF-67/MWCNT—Zeolitic imidazole framework/MWCNT, Poly (FSBF)MPGE—electro-polymerisation of fast sulphone black F on pencil graphite electrode, DPV—Differential pulse voltammetry, SWV—Square wave voltammetry, CV—Cyclic voltammetry.

## Data Availability

No data was used for the research described in the article.

## References

[1] Xue C., Wang X., Zhu W., Han Q., Zhu C., Hong J., Zhou X., Jiang H. (2014). Electrochemical Serotonin Sensing Interface Based on Double-Layered Membrane of Reduced Graphene Oxide/Polyaniline Nanocomposites and Molecularly Imprinted Polymers Embedded with Gold Nanoparticles. Sens. Actuators B Chem..

[2] Panneer Selvam S., Yun K. (2020). A Self-Assembled Silver Chalcogenide Electrochemical Sensor Based on RGO-Ag2Se for Highly Selective Detection of Serotonin. Sens. Actuators B Chem..

[3] Wang F., Wu Y., Lu K., Ye B. (2013). A Simple but Highly Sensitive and Selective Calixarene-Based Voltammetric Sensor for Serotonin. Electrochim. Acta.

[4] Han H.S., Lee H.K., You J.M., Jeong H., Jeon S. (2014). Electrochemical Biosensor for Simultaneous Determination of Dopamine and Serotonin Based on Electrochemically Reduced GO-Porphyrin. Sens. Actuators B Chem..

[5] Nataraj N., Chen T.W., Chen S.M., Tseng T.W., Bian Y., Sun T.T., Jiang J. (2021). Metal-Organic Framework (ZIF-67) Interwoven Multiwalled Carbon Nanotubes as a Sensing Platform for Rapid Administration of Serotonin. J. Taiwan Inst. Chem. Eng..

[6] Cesarino I., Galesco H.V., Machado S.A.S. (2014). Determination of Serotonin on Platinum Electrode Modified with Carbon Nanotubes/Polypyrrole/Silver Nanoparticles Nanohybrid. Mater. Sci. Eng. C.

[7] Wang Y., Wang S., Tao L., Min Q., Xiang J., Wang Q., Xie J., Yue Y., Wu S., Li X. (2015). A Disposable Electrochemical Sensor for Simultaneous Determination of Norepinephrine and Serotonin in Rat Cerebrospinal Fluid Based on MWNTs-ZnO/Chitosan Composites Modified Screen-Printed Electrode. Biosens. Bioelectron..

[8] Ran G., Chen C., Gu C. (2015). Serotonin Sensor Based on a Glassy Carbon Electrode Modified with Multiwalled Carbon Nanotubes, Chitosan and Poly(p-Aminobenzenesulfonate). Microchim. Acta.

[9] Santhan A., Hwa K.Y. (2023). Zinc Phosphate-Incorporated Niobium Carbide as an Effective Electrocatalyst for Ultrasensitive and Selective Monitoring of Monoamine Neurotransmitter. ACS Sustain. Chem. Eng..

[10] Xiao Y., Xiong C., Chen M.M., Wang S., Fu L., Zhang X. (2023). Structure Modulation of Two-Dimensional Transition Metal Chalcogenides: Recent Advances in Methodology, Mechanism and Applications. Chem. Soc. Rev..

[11] Jamal F., Rafique A., Moeen S., Haider J., Nabgan W., Haider A., Imran M., Nazir G., Alhassan M., Ikram M. (2023). Review of Metal Sulfide Nanostructures and Their Applications. ACS Appl. Nano Mater..

[12] Rajapriya A., Keerthana S., Viswanathan C., Ponpandian N. (2021). Direct Growth of MoS2 Hierarchical Nanoflowers on Electrospun Carbon Nanofibers as an Electrode Material for High-Performance Supercapacitors. J. Alloys Compd..

[13] Keerthana S., Rajapriya A., Viswanathan C., Ponpandian N. (2022). Hybrid Nanostructures of WS2 Nanoflowers on N, B Co-Doped RGO for Sensitive Amperometric Detection of Nilutamide. Mater. Today Chem..

[14] Wang J., Zhan P., Zhang D., Tang L. (2023). Nickel Cobalt Sulfide Composite Nanosheet Anchored on RGO as Effective Electrode for Quasi-Solid Supercapacitor. J. Energy Storage.

[15] Chanda D., Kannan K., Gautam J., Meshesha M.M., Jang S.G., Dinh V.A., Yang B.L. (2023). Effect of the Interfacial Electronic Coupling of Nickel-Iron Sulfide Nanosheets with Layer Ti3C2 MXenes as Efficient Bifunctional Electrocatalysts for Anion-Exchange Membrane Water Electrolysis. Appl. Catal. B Environ..

[16] Shi F., Zheng W., Wang W., Hou F., Lei B., Sun Z., Sun W. (2015). Application of Graphene-Copper Sulfide Nanocomposite Modified Electrode for Electrochemistry and Electrocatalysis of Hemoglobin. Biosens. Bioelectron..

[17] Bulakhe R.N., Sahoo S., Nguyen T.T., Lokhande C.D., Roh C., Lee Y.R., Shim J.J. (2016). Chemical Synthesis of 3D Copper Sulfide with Different Morphologies for High Performance Supercapacitors Application. RSC Adv..

[18] Roy P., Srivastava S.K. (2015). Nanostructured Copper Sulfides: Synthesis, Properties and Applications. CrystEngComm.

[19] Niu H., Liu Y., Mao B., Xin N., Jia H., Shi W. (2020). In-Situ Embedding MOFs-Derived Copper Sulfide Polyhedrons in Carbon Nanotube Networks for Hybrid Supercapacitor with Superior Energy Density. Electrochim. Acta.

[20] Zhang L., Chen M., Jiang Y., Chen M., Ding Y., Liu Q. (2017). A Facile Preparation of Montmorillonite-Supported Copper Sulfide Nanocomposites and Their Application in the Detection of H_2_O_2_. Sens. Actuators B Chem..

[21] Zhu J., Peng X., Nie W., Wang Y., Gao J., Wen W., Selvaraj J.N., Zhang X., Wang S. (2019). Hollow Copper Sulfide Nanocubes as Multifunctional Nanozymes for Colorimetric Detection of Dopamine and Electrochemical Detection of Glucose. Biosens. Bioelectron..

[22] Shamraiz U., Hussain R.A., Badshah A. (2016). Fabrication and Applications of Copper Sulfide (CuS) Nanostructures. J. Solid State Chem..

[23] Radhakrishnan S., Kim H.Y., Kim B.S. (2016). A Novel CuS Microflower Superstructure Based Sensitive and Selective Nonenzymatic Glucose Detection. Sens. Actuators B Chem..

[24] Kim W.B., Lee S.H., Cho M., Lee Y. (2017). Facile and Cost-Effective CuS Dendrite Electrode for Non-Enzymatic Glucose Sensor. Sens. Actuators B Chem..

[25] Sun S., Li P., Liang S., Yang Z. (2017). Diversified Copper Sulfide (Cu2-XS) Micro-/Nanostructures: A Comprehensive Review on Synthesis, Modifications and Applications. Nanoscale.

[26] Yuan C., Li N., Zhang X., Wang Y., Zhou S., Zhang L., Zhou M., Hu G. (2023). Flower-like Copper Sulfide-Decorated Boron-Nitrogen Co-Doped Carbon-Modified Glassy Carbon Electrode for Selective and Sensitive Electrochemical Detection of Nitrobenzene in Natural Water. Colloids Surf. A Physicochem. Eng. Asp..

[27] Chen Y., Sun Y., Waterhouse G.I.N., Gao H., Xu Z. (2023). Highly Selective Molecularly Imprinted Gel-Based Electrochemical Sensor with CuS@COOH-MWCNTs Signal Amplification for Simultaneous Detection of Vanillin and Tartrazine in Foods. Sens. Actuators B Chem..

[28] Tetyana P., Mphuthi N., Jijana A.N., Moloto N., Shumbula P.M., Skepu A., Vilakazi L.S., Sikhwivhilu L. (2023). Synthesis, Characterization, and Electrochemical Evaluation of Copper Sulfide Nanoparticles and Their Application for Non-Enzymatic Glucose Detection in Blood Samples. Nanomaterials.

[29] Peng J., Han X.X., Zhang Q.C., Yao H.Q., Gao Z.N. (2015). Copper Sulfide Nanoparticle-Decorated Graphene as a Catalytic Amplification Platform for Electrochemical Detection of Alkaline Phosphatase Activity. Anal. Chim. Acta.

[30] Zheng W., Yang Z., Qu W., Huang J., He W., Yang J., Yang W., Tian M., Xu Z., Li H. (2023). Mechanochemical Preparation of Well-Structured Copper Sulfide for Elemental Mercury Sequestration from Coal Combustion Flue Gas. Chem. Eng. J..

[31] Savarimuthu I., Susairaj M.J.A.M. (2022). CuS Nanoparticles Trigger Sulfite for Fast Degradation of Organic Dyes under Dark Conditions. ACS Omega.

[32] Tarachand, Hussain S., Lalla N.P., Kuo Y.K., Lakhani A., Sathe V.G., Deshpande U., Okram G.S. (2018). Thermoelectric Properties of Ag-Doped CuS Nanocomposites Synthesized by a Facile Polyol Method. Phys. Chem. Chem. Phys..

[33] Hurma T., Kose S. (2016). XRD Raman Analysis and Optical Properties of CuS Nanostructured Film. Optik.

[34] Shalabayev Z., Baláž M., Daneu N., Dutková E., Bujňáková Z., Kaňuchová M., Danková Z., Balážová Ĺ., Urakaev F., Tkáčiková Ĺ. (2019). Sulfur-Mediated Mechanochemical Synthesis of Spherical and Needle-Like Copper Sulfide Nanocrystals with Antibacterial Activity. ACS Sustain. Chem. Eng..

[35] Samdhyan K., Chand P., Anand H. (2023). Effective Doping of Phosphorus in Copper Sulfide for High Performance Energy Storage Devices. J. Alloys Compd..

[36] Dang J., Yin M., Pan D., Tian Z., Chen G., Zou J., Miao H., Wang Q., Yuan J. (2023). Four-Functional Iron/Copper Sulfide Heterostructure for Alkaline Hybrid Zinc Batteries and Water Splitting. Chem. Eng. J..

[37] Nataraj N., Chen T.W., Gan Z.W., Chen S.M., Lou B.S., Ali M.A., Al-Hemaid F.M. (2022). Two-Dimensional Copper Oxide/Zinc Oxide Nanoflakes with Three-Dimensional Flower-like Heterostructure Enhanced with Electrocatalytic Activity toward Nimesulide Detection. Mater. Today Chem..

[38] Santhan A., Hwa K.Y. (2022). Rational Design of Nanostructured Copper Phosphate Nanoflakes Supported Niobium Carbide for the Selective Electrochemical Detection of Melatonin. ACS Appl. Nano Mater..

[39] Das M., Das D., Sil S., Ray P.P. (2023). Development of Hierarchical Copper Sulfide–Carbon Nanotube (CuS–CNT) Composites and Utilization of Their Superior Carrier Mobility in Efficient Charge Transport towards Photodegradation of Rhodamine B under Visible Light. Nanoscale Adv..

[40] Song Z., Liu Y., Zhang B., Song S., Zhou Z., Huang Y., Zhao Z. (2023). Magnetic Grinding Synthesis of Copper Sulfide-Based Photocatalytic Composites for the Degradation of Organic Dyes under Visible Light. New J. Chem..

[41] Xiao Y., Huang Y., Cheng H., Wu J., Jin B. (2023). Development of Copper Sulfide Functionalized CeO2 Nanoparticle for Strengthened Removal of Gaseous Elemental Mercury from Flue Gas. Chem. Eng. J..

[42] Zheng L., Chen G., Huang J., Chen W., Han T., Li T., Ken Ostrikov K. (2024). Oxygen Evolution Catalyzed by Ni-Co-Nb Ternary Metal Sulfides on Plasma-Activated Ni-Co Support. J. Colloid Interface Sci..

[43] Shahid M.M., Rameshkumar P., Numan A., Shahabuddin S., Alizadeh M., Khiew P.S., Chiu W.S. (2019). A Cobalt Oxide Nanocubes Interleaved Reduced Graphene Oxide Nanocomposite Modified Glassy Carbon Electrode for Amperometric Detection of Serotonin. Mater. Sci. Eng. C.

[44] Khoshnevisan K., Baharifar H., Torabi F., Sadeghi Afjeh M., Maleki H., Honarvarfard E., Mohammadi H., Sajjadi-Jazi S.M., Mahmoudi-Kohan S., Faridbod F. (2021). Serotonin Level as a Potent Diabetes Biomarker Based on Electrochemical Sensing: A New Approach in a Zebra Fish Model. Anal. Bioanal. Chem..

[45] Banu R., Swamy B.E.K., Deepa S. (2020). Poly (Fast Sulphone Black F) Modified Pencil Graphite Electrode Sensor for Serotonin. Sens. Int..

